# The comparison of risk factors for colorectal neoplasms at different anatomical sites

**DOI:** 10.1007/s00384-022-04296-3

**Published:** 2023-01-31

**Authors:** Huaqing Wang, Zhen Yuan, Shuyuan Wang, Wenwen Pang, Wanting Wang, Xinyu Liu, Ben Yi, Qiurong Han, Yao Yao, Qinghuai Zhang, Xipeng Zhang, Chunze Zhang

**Affiliations:** 1 Department of Colorectal Surgery, Tianjin Union Medical Center, Tianjin, China; 2 School of Medicine, Nankai University, Tianjin, China; 3 School of Integrative Medicine, Tianjin University of Traditional Chinese Medicine, Tianjin, China; 4 Tianjin Medical University, Tianjin, China; 5 Department of Clinical Laboratory, Tianjin Union Medical Center, Tianjin, China; 6 The Institute of Translational Medicine, Tianjin Union Medical Center, Tianjin, China; 7 Tianjin Institute of Coloproctology, Tianjin, China

**Keywords:** Colorectal neoplasms, Different anatomical sites, Risk factors, Colorectal adenoma, Colorectal cancer

## Abstract

**Aim:**

Both the clinical manifestation and molecular characteristics of colorectal cancer (CRC) vary according to the anatomical site. We explored the risk factors for four groups of colorectal neoplasms (CRN) at different anatomical sites.

**Methods:**

We extracted data from the database of Tianjin Colorectal Cancer Screening Program from 2010 to 2020. According to the CRN anatomical sites, patients were divided into four groups: the proximal colon group, the distal colon group, the rectum group, and the multiple colorectal sites. Binary logistic regression analysis was used to explore the differences in risk factors of CRN at different anatomical sites.

**Results:**

The numbers of patients with CRN in the proximal colon, distal colon, rectum, and multiple colorectal sites were 4023, 6920, 3657, and 7938, respectively. Male sex was associated with a higher risk from the proximal colon to the rectum. Advanced age and obesity were also significantly associated with overall colorectal CRN risk, but there were some differences between men and women. Smoking was associated with CRN risk only in the distal colon and rectum in both men and women. Frequent alcohol consumption and family history of CRC in first-degree relatives (FDRs) were associated with the risk of multisite colorectal CRN only in males.

**Conclusions:**

We observed differences in advanced age, obesity, smoking, alcohol consumption, and family history of colorectal cancer at different anatomical sites of colorectal neoplasms. These factors vary by gender.

## Introduction

Colorectal cancer is the third most common cancer in the world and the second most common cancer in the world [1]. According to the latest statistics in 2022, colorectal cancer is the second most common cancer and the fifth leading cause of cancer death in China [2]. Adenoma is considered a precancerous lesion[3]. Most colorectal cancers are transformed from preexisting adenomas over many years. The adenoma-carcinoma sequence may involve the activation of multiple oncogenes by mutation and the inactivation of multiple tumor suppressor genes [4], a progressive progression from normal tissue to abnormal tissue to cancer [5]. Therefore, early screening for colorectal cancer can prevent and diagnose colorectal cancer. High-risk adenomas can be detected and removed by colonoscopy, thereby reducing the incidence and mortality of colorectal cancer [6].

Embryologic development from the proximal and distal colon differs in origin, with the portion from the cecum to the proximal two-thirds of the transverse colon originating in the midgut and the portion from the distal transverse colon to the rectum originating in the hindgut [7]. In 1990, Bufill proposed that proximal colon cancer and distal colon cancer were two distinct tumors and proved this from a molecular genetic point of view [8]. Later, some scholars explored the risk factors for colorectal cancer in different locations [9, 10]. However, there are few studies to analyze the risk factors of colorectal neoplasms (CRN) (colorectal adenoma and cancer) at different sites. Approximately 5% of adenomas become malignant within 5 to 10 years [4], so adenoma is also a dangerous disease. Our screening program was designed to detect CRN in a timely manner, so as to intervene early in the transformation of adenoma to cancer or make preventive recommendations. Therefore we will comprehensively study the difference in risk factors between colorectal adenoma and colorectal cancer in different sites.

In this study, we divided colorectal neoplasms into the proximal colon group, distal colon group, rectal group, and multiple colorectal site groups (whole colon or colorectal) according to their anatomical sites. Exploring the risk factors of colorectal neoplasms in different anatomical sites is of great significance for colorectal cancer screening, which can guide people to prevent the occurrence of colorectal neoplasms by changing their lifestyle, and ultimately plays a role in preventing colorectal cancer and reducing the incidence and mortality of colorectal cancer.

## Materials and methods

### Data source

Since 2010, the Tianjin municipal government has organized and commissioned the Tianjin Union Medical Center and other community hospitals to conduct colorectal cancer screening. Participants completed a questionnaire for colorectal cancer risk factors or underwent a stool immunochemical test, and those who were positive for either were subjected to colonoscopy. The population in this study was part of the colorectal Cancer Screening Program in Tianjin. All participants signed an informed consent form. This study was approved by the Ethics Committee of Tianjin Union Medical Center. This trial was conducted following the Declaration of Helsinki guidelines.

### Colonoscopy procedures and definition of CRN

All colonoscopies are performed by experienced physicians at the hospital who have undergone formal training, rigorous examination, and certification. Good bowel preparation was performed for each colonoscopy, and a clear photographic record was maintained for adequate examination time. All pathological findings were compared and reported by a physician specializing in pathology against the latest guidelines. Colonic pathologic findings included colorectal cancer, advanced adenomas (≥ 1.0 cm in diameter or with villous components or pathological findings of high-grade intraepithelial neoplasia), non-advanced adenoma, ulcerative colitis, Crohn’s disease, benign tumor, and non-adenomatous polyps. CRN include carcinoma, advanced adenoma, and non-advanced adenoma.

### Select study participants

We first screened participants who were positive on the high-risk questionnaire or fecal immunochemical test. Those who meet any or more of the following criteria are considered to be at high risk, and those at high risk are advised to undergo colonoscopy to confirm the diagnosis: (1) first-degree relatives with colorectal cancer; (2) personal history of malignant tumors or intestinal polyps; (3) patients with two or more of the following: (a) chronic constipation, (b) chronic diarrhea, (c) mucinous stool or bloody stool, (d) history of adverse life events, (e) history of chronic appendicitis or appendectomy, and (f) history of chronic cholecystitis or cholecystectomy; and (4) positive fecal occult blood test.

Participants with successful colonoscopy and complete pathological reports were included, and benign tumors, ulcerative colitis, Crohn’s disease, and non-adenomatous polyps were excluded. Participants with incomplete questionnaires were also excluded.

### Classification of anatomical sites of CRN

According to the site of CRN, we divided the participants into four groups: proximal colon group, distal colon group, rectum group, and multisite colorectal site group. The proximal colon group included the cecum, ascending colon, colonic liver curvature, and transverse colon. The distal colon group included the splenic curvature, descending colon, and sigmoid colon. The rectum group included the rectosigmoid junction. Whole colon or multiple colorectal groups were set to explore which exposure factors promote the development of adenomas in multiple anatomical sites within the colorectum.

### Assessment of exposure factors

We collated data from the questionnaire, including sex, age, lesion site, pathology, smoking status, frequency of alcohol consumption, body mass index (BMI), and history of colorectal cancer in first-degree relatives (FDRs). Age was divided into four groups (40–50, 51–60, 61–70, and older than 70). Smoking status was divided into two groups (never, present, or past). Drinking frequency was divided into two groups (never or occasionally, weekly, or daily). According to the criteria of the Asian population recommended by the WHO, the critical point of BMI for overweight and obesity is defined as 23 kg/m^2^ and 27.5 kg/m^2^, respectively [11].

### Statistical analysis

All statistical analyses were performed using SPSS software version 26.00. The number and proportion of patients with CRN features at each site were calculated. The independent risk factors of colorectal adenoma in different anatomical sites were analyzed by dichotomous logistic regression. Univariate analysis and multivariate analysis were performed, and *P* < 0.05 was statistically significant by two-sided test. The odds ratio (OR) and 95% confidence interval (95% CI) of each variable were calculated.

## Results

### Characteristics of participants

**Fig. 1:**
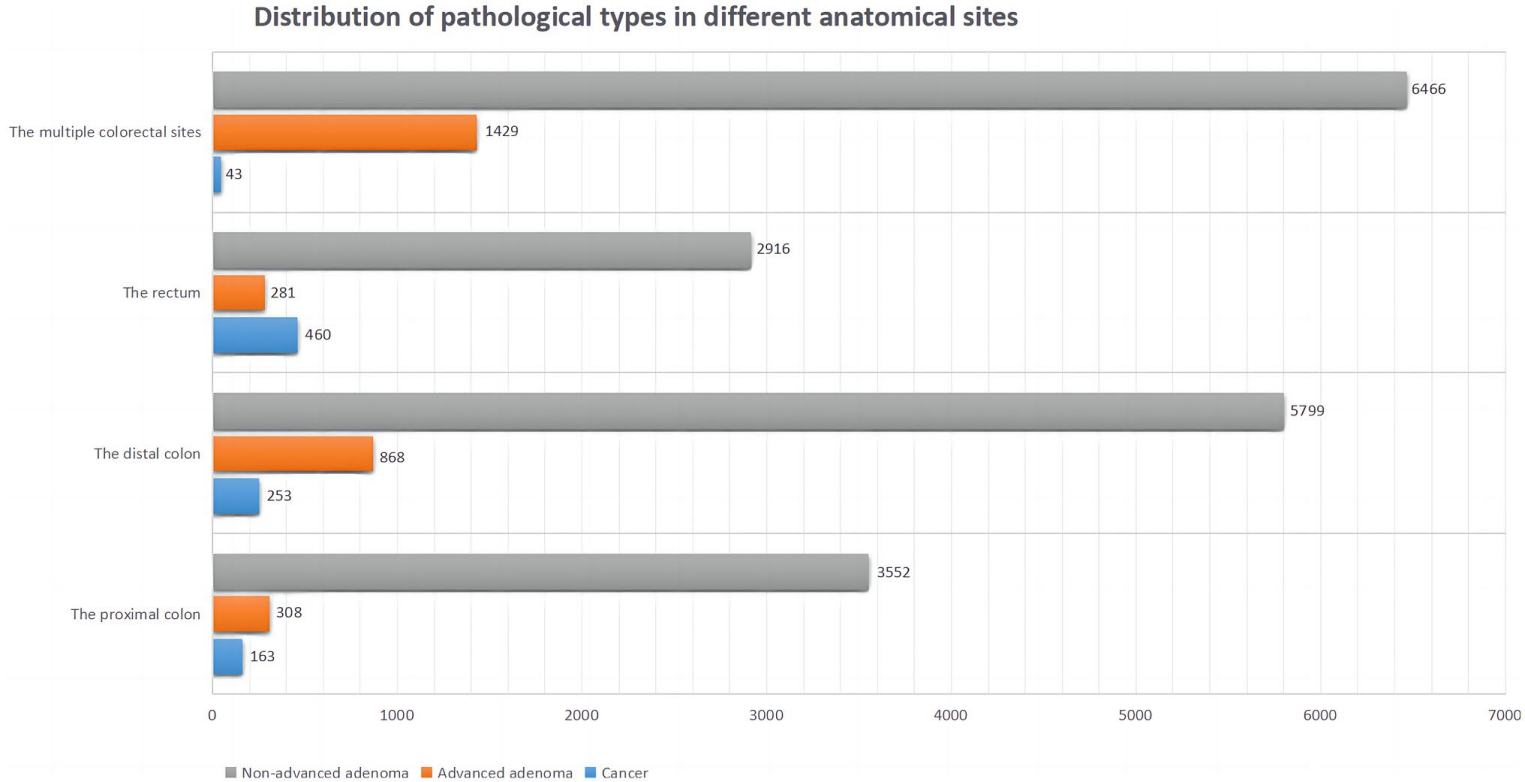
Distribution of carcinoma, advanced adenoma, and non-advanced adenoma in different groups

A total of 41,352 participants were enrolled in this study, of whom 22,538 (54.5%) were diagnosed with CRN, including 919 (2.2%) with CRC, 18,733 (45.3%) with non-advanced adenoma, and 2866 (6.9%) with advanced adenoma. Among all patients diagnosed with CRN, 9945 (44.1%) were females and 12,593 (55.9%) were males. There were 4023 cases (17.8%) in the proximal colon group, 6920 cases (30.7%) in the distal colon group, 3657 cases (16.2%) in the rectum group, and 7938 cases (35.2%) in the whole colon or colorectal group. Table 1 shows the basic characteristics of all participants. Figure 1 shows the distribution of pathological types of CRN in different sites. The percentages of non-advanced adenoma, advanced adenoma, and cancer in each group were as follows: proximal colon group (88.3%, 7.7%, 4.1%), distal colon group (83.8%, 12.5%, 3.7%), rectum group (79.7%, 7.7%, 12.6%), and multi-site colorectal group (81.5%, 18.0%, 0.5%).

**Table 1:** Characteristics of the 41,352 participants

**Variables**	**Participants without CRN**	**Participants with CRN**			
		**The proximal colon**	**The distal colon**	**The rectum**	**The multiple colorectal sites**
	**(*n*** **= 18,814)**	**(*n*** **= 4023)**	**(*n*** **= 6920)**	**(*n*** **= 3657)**	**(*n*** **= 7938)**
**Sex (%)**					
Female	11,669 (62.0)	2038 (50.7)	3120 (45.1)	1800 (49.2)	2987 (37.6)
Male	7145 (38.0)	1985 (49.3)	3800 (54.9)	1857 (50.8)	4951 (62.4)
**Age (years) (%)**					
40–50	1943 (10.3)	230 (5.7)	436 (6.3)	236 (6.5)	276 (3.5)
51–60	5813 (30.9)	1170 (29.1)	2123 (30.7)	904 (24.7)	1506 (19.0)
61–70	8400 (44.6)	1975 (49.1)	3372 (48.7)	1818 (49..7)	4247 (53.5)
Greater than 70	2658 (14.1)	648 (16.1)	989 (14.3)	699 (19.1)	1909 (24.0)
**BMI (kg/m**^2^**) (%)**					
Less than 23	6013 (32.0)	1087 (27.0)	1899 (27.4)	1041 (28.5)	1904 (24.0)
23–27.5	9714 (51.6)	2177 (54.1)	3672 (53.1)	1970 (53.9)	4260 (53.7)
Greater than 27.5	3087 (16.4)	759 (18.9)	1349 (19.5)	646 (17.7)	1774 (22.3)
**Smoking status (%)**					
Never	15,541 (82.6)	3189 (79.3)	4960 (71.7)	2736 (74.8)	5347 (67.4)
Present or past	3273 (17.4)	834 (20.7)	1960 (28.3)	921 (25.2)	2591 (32.6)
**Alcohol intake (%)**					
Never or occasional	17,424 (92.6)	3628 (90.2)	6063 (87.6)	3248 (88.8)	6670 (84.0)
Weekly or daily	1390 (7.4)	395 (9.8)	857 (12.4)	409 (11.2)	1268 (16.0)
**Family history of CRC (%)**					
No	17,331 (92.1)	3668 (91.2)	6370 (92.1)	3368 (92.1)	7270 (91.6)
Yes	1483 (7.9)	355 (8.8)	550 (7.9)	289 (7.9)	668 (8.4)
**Regular exercise (%)**					
Yes	7885 (41.8)	1795 (44.6)	2993 (43.3)	1660 (45.4)	3880 (48.9)
No	10,949 (58.2)	2228 (55.4)	3927 (56.7)	1997 (54.6)	4058 (51.1)
**History of chronic diarrhea (%)**					
No	15,065 (80.1)	3338 (83.0)	5769 (83.4)	2955 (80.8)	6610 (83.3)
Yes	3749 (19.9)	685( 17.0)	1151 (16.6)	702 (19.2)	1328 (16.7)
**History of chronic constipation (%)**					
No	14,855 (79.0)	3266 (81.2)	5718 (82.6)	3004 (82.1)	6578 (82.9)
Yes	3959 (21.0)	757 (18.8)	1202 (17.4)	653 (17.9)	1360 (17.0)
**History of mucous and bloody stool (%)**					
No	16,199 (86.1)	3514 (87.3)	6068 (87.7)	3110 (85.0)	6983 (88.0)
Yes	2615 (13.9)	509 (12.7)	852 (12.3)	547 (15.0)	955 (12.0)

Family history of colorectal cancer was defined only in first-degree relatives

*CRN colorectal neoplasms, *CRC* colorectal cancer, *BMI* body mass index

### Univariate analysis and multivariate analysis

**Fig. 2:**
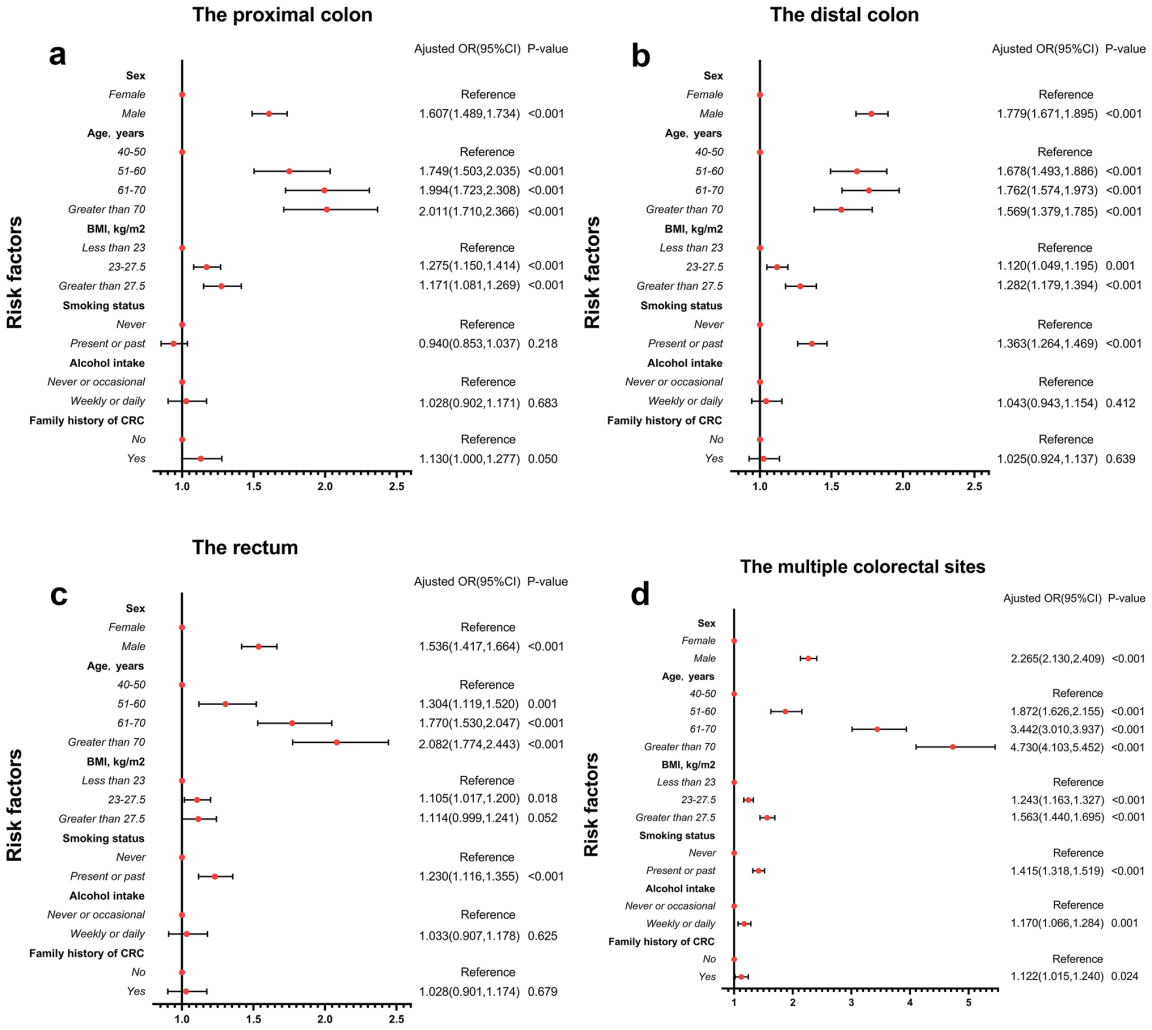
Forest plots used to show odds ratios for CRN risk factors in different groups. **a** The proximal colon subgroup. **b** The distal colon subgroup. **c** The rectum subgroup. **d** The multiple colorectal sites subgroup. *CRC colorectal cancer, BMI body mass index, OR odds ratio, CI confidence interval. Family history of colorectal cancer was defined only in first-degree relatives

Table 2 shows the results of univariate logistic regression analysis. Male sex, advanced age, overweight or obesity, smoking in the past or present, and drinking multiple times per week were risk factors significantly associated with CRN in the proximal colon, distal colon, rectum, and multiple sites of colorectum. A history of colorectal cancer in a first-degree relative was only a risk factor for proximal colon CRN. Figure 2 shows the results of multivariate logistic regression analysis, and Table 3 shows the differences between men and women after stratification:

**Table 2:** Univariate logistic regression analysis was used to analyze the risk factors of CRN at different anatomical sites

**Risk factors**	**The proximal colon**			**The distal colon**		**The rectum**		**The multiple colorectal sites**	
	**Unadjusted OR (95% CI)**		***P*** **value**	**Unadjusted OR (95% CI)**	***P*** **value**	**Unadjusted OR (95% CI)**	***P*** **value**	**Unadjusted OR (95% CI)**	***P*** **value**
**Sex**									
Female	Reference			Reference		Reference		Reference	
Male	1.591 (1.485, 1.7032)		< 0.001	1.989 (1.881, 2.103)	< 0.001	1.685 (1.569, 1.809)	< 0.001	2.707 (2.564, 2.858)	< 0.001
**Age (years)**									
40–50	Reference			Reference		Reference		Reference	
51–60	1.700 (1.463, 1.976)		< 0.001	1.628 (1.451, 1.826)	< 0.001	1.280 (1.100, 1.491)	0.001	1.824 (1.588, 2.094)	< 0.001
61–70	1.986 (1.718, 2.297)		< 0.001	1.789 (1.601, 2.000)	< 0.001	1.782 (1.542, 2.058)	< 0.001	3.559 (3.121, 4.059)	< 0.001
Greater than 70	2.060 (1.753, 2.420)		< 0.001	1.658 (1.460, 1.883)	< 0.001	2.165 (1.847, 2.538)	< 0.001	5.056 (4.399, 5.811)	< 0.001
**BMI (kg/m**^2^**)**									
Less than 23	Reference			Reference		Reference		Reference	
23–27.5	1.240 (1.145, 1.342)		< 0.001	1.197 (1.123, 1.276)	< 0.001	1.171 (1.080, 1.271)	< 0.001	1.385 (1.301, 1.475)	< 0.001
Greater than 27.5	1.360 (1.228, 1.507)		< 0.001	1.384 (1.275, 1.502)	< 0.001	1.209 (1.086, 1.346)	0.001	1.815 (1.679, 1.962)	< 0.001
**Smoking status**									
Never	Reference			Reference		Reference		Reference	
Present or past	1.242 (1.141, 1.352)		< 0.001	1.8776 (1.759, 2.001)	< 0.001	1.598 (1.470, 1.738)	< 0.001	2.301 (2.166, 2.444)	< 0.001
**Alcohol intake**									
Never or occasional	Reference			Reference		Reference		Reference	
Weekly or daily	1.365 (1.214, 1.535)		< 0.001	1.772 (1.619, 1.939)	< 0.001	1.578 (1.405, 1.773)	< 0.001	2.383 (2.197, 2.585)	< 0.001
**Family history of CRC**									
No	Reference			Reference		Reference		Reference	
Yes	1.131 (1.002, 1.277)	0.046		1.009 (0.911, 1.117)	0.863	1.003 (0.879, 1.144)	0.967	1.074 (0.976, 1.181)	0.143

Family history of colorectal cancer was defined only in first-degree relatives

*CRN colorectal neoplasms, *CRC* colorectal cancer, *BMI* body mass index, *OR* odds ratio, *CI* confidence interval

**Table 3:** Risk factors for CRN at different anatomical sites stratified by sex

**Risk factors**	**The proximal colon**		**The distal colon**		**The rectum**		**The multiple colorectal sites**	
	**Adjusted OR (95% CI)**	***P*** **value**	**Adjusted OR (95% CI)**	***P*** **value**	**Adjusted OR (95% CI)**	***P*** **value**	**Adjusted OR (95% CI)**	***P*** **value**
**Age (years)**								
**Female**								
40–50	Reference		Reference		Reference		Reference	
51–60	1.822 (1.455, 2.280)	< 0.001	1.646 (1.380, 1.965)	< 0.001	1.099 (0.883, 1.368)	0.397	1.700 (1.347, 2.146)	< 0.001
61–70	2.111 (1.695, 2.629)	< 0.001	1.806 (1.520, 2.147)	< 0.001	1.684 (1.368, 2.074)	< 0.001	3.431 (2.745, 4.289)	< 0.001
Greater than 70	2.466 (1.938, 3.139)	< 0.001	1.764 (1.450, 2.145)	< 0.001	2.051 (1.629, 2.584)	< 0.001	5.404 (4.277, 6.828)	< 0.001
**Male**								
40–50	Reference		Reference		Reference		Reference	
51–60	1.720 (1.399, 2.115)	< 0.001	1.730 (1.479, 2.023)	< 0.001	1.549 (1.250, 1.920)	< 0.001	2.032 (1.701, 2.428)	< 0.001
61–70	1.913 (1.570, 2.331)	< 0.001	1.713 (1.474, 1.992)	< 0.001	1.818 (1.482, 2.228)	< 0.001	3.435 (2.903, 4.065)	< 0.001
Greater than 70	1.658 (1.328, 2.068)	< 0.001	1.402 (1.181, 1.664)	< 0.001	2.068 (1.656, 2.583)	< 0.001	4.184 (3.497, 5.005)	< 0.001
**BMI (kg/m**^2^**)**								
** Female**								
Less than 23	Reference		Reference		Reference		Reference	
23–27.5	1.112 (0.999, 1.238)	0.052	1.133 (1.035, 1.241)	0.007	1.125 (1.004, 1.260)	0.042	1.306 (1.185, 1.439)	< 0.001
Greater than 27.5	1.207 (1.049, 1.387)	0.008	1.303 (1.160, 1.465)	< 0.001	1.161 (1.001, 1.348)	0.049	1.599 (1.419, 1.801)	< 0.001
**Male**								
Less than 23	Reference		Reference		Reference		Reference	
23–27.5	1.249 (1.106, 1.410)	< 0.001	1.104 (1.005, 1.213)	0.040	1.079 (0.956, 1.218)	0.217	1.187 (1.085, 1.299)	< 0.001
Greater than 27.5	1.356 (1.162, 1.582)	< 0.001	1.253 (1.111, 1.414)	< 0.001	1.058 (0.903, 1.241)	0.485	1.514 (1.353, 1.694)	< 0.001
**Smoking status**								
**Female**								
Never	Reference		Reference		Reference		Reference	
Present or past	1.078 (0.880, 1.320)	0.467	1.707 (1.470, 1.983)	< 0.001	1.483 (1.227, 1.794)	< 0.001	1.803 (1.558, 2.087)	< 0.001
**Male**								
Never	Reference		Reference		Reference		Reference	
Present or past	0.897 (0.803, 1.002)	0.053	1.262 (1.158, 1.375)	< 0.001	1.147 (1.026, 1.283)	0.016	1.298 (1.198, 1.407)	< 0.001
**Alcohol intake**								
**Female**								
Never or occasional	Reference		Reference		Reference		Reference	
Weekly or daily	1.490 (0.863, 2.572)	0.152	1.083 (0.659, 1.779)	0.753	0.958 (0.499, 1.839)	0.897	1.460 (0.935, 2.28)	0.096
**Male**								
Never or occasional	Reference		Reference		Reference		Reference	
Weekly or daily	1.031 (0.900, 1.181)	0.656	1.079 (0.972, 1.198)	0.155	1.075 (0.938, 1.230)	0.298	1.202 (1.092, 1.323)	< 0.001
**Family history of CRC**								
**Female**								
No	Reference		Reference		Reference		Reference	
Yes	1.121 (0.953, 1.319)	0.168	1.015 (0.880, 1.170)	0.839	1.126 (0.947, 1.339)	0.178	1.068 (0.924, 1.236)	0.373
**Male**								
No	Reference		Reference		Reference		Reference	
Yes	1.147 (0.953, 1.381)	0.147	1.049 (0.901, 1.221)	0.541	0.918 (0.747, 1.128)	0.415	1.182 (1.028, 1.359)	0.019

Family history of colorectal cancer was defined only in first-degree relatives

*CRN colorectal neoplasms, *CRC* colorectal cancer, *BMI* body mass index, *OR* odds ratio, *CI* confidence interval

#### Sex and age

Male sex was an independent risk factor for CRN from the proximal colon to the rectum. Males had the strongest correlation with CRN risk in the distal colon (OR = 1.779, 95% CI = 1.671, 1.895). The risk of CRN increased with age in both the proximal colon and rectum. For all sexes, age over 70 conferred the greatest risk in the proximal colon. The risk of CRN was greatest in the distal colon at 61–70 years of age (OR = 1.762, 95% CI = 1.574, 1.973). For women, age over 70 years (OR = 2.466, 95% CI = 1.938, 3.139) was most strongly associated with CRN risk in the proximal colon. However, for men, age over 70 years (OR = 2.068, 95% CI = 1.656, 2.583) was most strongly associated with rectal CRN risk. In the colon, older age was associated with a higher risk of CRN in women than in men. However, advanced age in the rectum confers a greater risk to men.

#### BMI

Obesity (BMI greater than 27.5 kg/m^2^) was associated with CRN risk from the proximal colon to the rectum. For women, obesity (OR = 1.303, 95% CI = 1.160, 1.465) conferred the greatest risk in the distal colon. For men, however, obesity (OR = 1.356, 95% CI = 1.162, 1.582) conferred the greatest risk in the proximal colon. There is no significant correlation between obesity and the increased risk of rectal CRN in men.

#### Unhealthy lifestyle and family history of CRC (FDRs)

Multivariate analysis showed that smoking was only associated with CRN in the distal colon (OR = 1.363, 95% CI = 1.264, 1.469) and rectum (OR = 1.230, 95% CI = 1.116, 1.355). And the risk is greater in the distal colon. This is true for all sexes. In the distal colon, for women, the OR for smoking is 1.707 (95% CI = 1.470, 1.983); for men, the OR for smoking is 1.262 (95% CI = 1.158, 1.375). Among CRN patients with single intestinal segment, we found that multiple alcohol consumption per week and family history of CRC were not significantly associated with CRN occurrence, but were only associated with CRN at multiple sites. Further analysis in men and women revealed that these two factors were surprisingly only associated with the incidence of colorectal multisite CRN in men. Among men with colorectal multisite CRN, the OR for frequent drinking is 1.202 (95% CI = 1.092, 1.323), and the OR for family history of CRC (FDRs) is 1.182 (95% CI = 1.028, 1.359).

Notably, older age, obesity, and smoking were more associated with multiple-site CRN than with a single bowel segment in both men and women. But this group also had the largest proportion of non-advanced adenomas and advanced adenomas.

## Discussion

Male sex and advanced age were significantly associated with CRN risk in any part of the colorectal region: males had the strongest association with CRN in the distal colon. Age over 70 years was most associated with the risk of CRN in the proximal colon among women and rectal CRN among men. Men are more likely to develop CRN than women, possibly because men lack the protective effect of estrogen [12]. Moreover, it has been suggested that androgens may be involved in the formation of colorectal tumors by regulating the proliferation of intestinal epithelial cells [13]. By comparison, we found that only when CRN occurred in the proximal colon did the proportion of women exceed that of men, which is consistent with previous findings that women are at higher risk for CRC in the proximal colon [14], especially in the ascending colon [9]. In many studies, advanced age is a risk factor for colorectal adenoma and colorectal cancer [9, 12, 15, 16]. This may be related to the adenoma-carcinoma sequence of colorectal tumors [5, 17]; when normal cells accumulate multiple genetic mutations over many years, they become cancerous [18, 19]. Therefore, early detection of high-risk adenomas is also one of the goals of our screening program, and the progression of colorectal neoplasms can be cut off by interfering with the adenoma-carcinoma sequence of CRC [20].

Obesity has repeatedly been shown to be a risk factor for colorectal adenoma, colorectal cancer, and recurrence after adenoma resection [19, 21]. In Asian populations, 23 kg/m^2^ and 27.5 kg/m^2^ are the latest recommended cut-off values for overweight and obesity, respectively, which are both risk factors for CRN from the proximal colon to the rectum, when not stratified by sex. However, there was no significant association between obesity and rectal CRN risk in men. For multiple colorectal CRN, high BMI was significantly more associated with CRN risk than single intestinal segment, which may indicate that obesity is a risk factor without anatomical heterogeneity, and obesity may increase the risk of CRN in both the colon and rectum indiscriminately. Racial and ethnic differences may influence obesity-related differences in CRN risk across colorectal sites [21, 22].

Smoking and MSI-H, CIMP (CpG island methylator phenotype), and a mutated BRAF gene are related [23], and the frequency of these three genetic changes from the rectum to the ascending colon increases gradually [24], which may be the molecular basis for the different associations between smoking and CRN risk at different sites. We found that smoking was a risk factor for CRN in the distal colon, rectum, and multiple colorectal sites, but not in the proximal colon. However, it was previously reported that smoking was most strongly associated with the risk of CRC in the proximal colon (primarily the transverse colon) and rectum [9, 25]. Some studies have suggested that smoking may have a greater effect on non-advanced adenomas, and our study included a large proportion of patients with colorectal adenomas, which may have contributed to the difference in results [26]. Smoking is more associated with CRN risk in women, which has been confirmed in other studies[27]. But we do not come to the same conclusion. Although the odds ratio for smoking was higher in women than in men, it is not sufficient to conclude that smoking is more harmful to women, because men are at higher risk for CRN, which may also contribute to the difference in this data. Although smoking mainly causes malignant tumors of the respiratory system, it also increases the risk of developing tumors of the digestive and urinary systems [28]. Smoking generates a variety of carcinogenic compounds through the digestive system, circulatory system, and intestinal contact; causes intestinal flora metabolic disorders; and induces the generation of adenoma and progression to CRC [29]. Quitting smoking can limit the progression of CIMP [22], which has guiding significance for the prevention of CRC.

Alcohol also increases the risk of CRC by causing DNA damage and dysregulation of cellular REDOX reactions through the production of acetaldehyde and acetate by metabolism in the body [30]. We found that although frequent alcohol consumption was associated with CRN risk at all sites in univariate analysis, it was only an independent risk factor in males for colorectal multisite CRN in multivariate analysis. Previous studies are consistent with our results that alcohol consumption only poses a risk for men [31]. Alcohol consumption has been shown to be a risk factor for colorectal adenoma [32], but the association with colorectal cancer is weaker [33]. In our study, the proportions of non-advanced and advanced adenomas in the colorectal multisite group were 34.5% and 49.5%, respectively. Therefore, at this point, our results may mainly show the risk of colorectal adenomas.

We found that a history of colorectal cancer in a first-degree relative was an independent risk factor for CRN in the proximal colon and multisite colorectal sites. For all sexes, it was only a marginal risk factor for the proximal colon. However, when stratified by sex, it was only associated with the risk of multisite CRN in males. Many studies have shown that people with a family history of colorectal cancer are more likely to develop CRN [34–37]. The American College of Gastroenterology guidelines for colorectal cancer screening define those with colorectal cancer in first-degree relatives as at high risk and recommend colonoscopy starting at age 40 [6]. Other researchers have reached similar conclusions: A family history of colorectal cancer is most strongly associated with the risk of proximal colon cancer or adenoma [38, 39]. Family history of CRC may be associated with most molecular subtypes, and some types of colorectal cancer contain multiple molecular subtypes, for example, LINE-1 (long interspersed nucleotide element 1) methylation and CIMP-low of MSI-high cancer. These molecular isoforms will change along the gut [24, 40]. Moreover, MSI-high tumors are more likely to occur in the proximal colon [41], which may underlie the anatomical heterogeneity of CRC family history.

There are some limitations in our study. First, the classification of anatomical sites is not sufficiently refined, and some potential variations may be obscured by broad divisions. Second, there may be recall bias in the contents of the questionnaire. Third, there are many bad living habits, and we only considered some of them. Fourth, some participants were excluded because of incomplete pathology reports or questionnaire content, which may have interfered with the results.

In conclusion, we explored risk factors for CRN based on anatomical sites. Men are an immutable risk factor. Advanced age increases the risk of CRN, but the sites with the strongest correlation differ by sex. Obesity was associated with CRN risk in both men and women, but was not significantly associated with an increased risk of rectal CRN in men. Smoking is more harmful to women. Alcohol consumption and family history of colorectal cancer contributed less to CRN than other factors. Recognition of these differences will guide the development of population-specific prevention and treatment strategies in colorectal cancer screening programs.

## Data Availability

The datasets used and analyzed during the current study are available from the corresponding author on reasonable request.
